# Copper and Its Complexes in Medicine: A Biochemical Approach

**DOI:** 10.4061/2011/594529

**Published:** 2011-06-15

**Authors:** Isidoros Iakovidis, Ioannis Delimaris, Stylianos M. Piperakis

**Affiliations:** ^1^Department of Physics Chemistry & Materials Technology, Technological Educational Institute of Athens, 12210 Athens, Greece; ^2^Biology Unit, Department of Pre-School Education, University of Thessaly, Argonafton and Filellinon Streets, Volos 38221, Greece

## Abstract

The fundamental role of copper and the recognition of its complexes as important bioactive compounds *in vitro* and *in vivo* aroused an ever-increasing interest in these agents as potential drugs for therapeutic intervention in various diseases. The vast array of information available for their bioinorganic properties and mode of action in several biological systems, combined with the new opportunities offered by the flourishing technologies of medicinal chemistry, is creating an exciting scenario for the development of a novel generation of highly active drugs with minimized side effects which could add significantly to the current clinical research and practice. In this paper we attempt to summarize all the available-to-date information on these issues.

## 1. Introduction

Copper exhibits considerable biochemical action either as an essential trace metal or as a constituent of various exogenously administered compounds in humans. In its former role it is bound to ceruloplasmin, albumin, and other proteins, while in its latter it is bound to ligands of various types forming complexes that interact with biomolecules, mainly proteins and nucleic acids. The multifaceted role of copper in biological systems is demonstrated by several studies. In particular the involvement of copper in human diseases has been described from a medicinal-chemical [1] and a biochemical view [2] focusing on the molecular physiology of Cu transport [3]. Much of the current research effort is cited on copper homeostasis [4] and its relation to iron metabolism [5] as well as the role of copper in biological processes related to human physiology and pathology [6, 7]. While a lot of the functions that have been proposed to account for the homeostasis of inorganic noncomplexed copper in humans have been described [3–5], only a limited number of review studies have focused on the multiple biochemical events which could be directly implicated in the use of copper complexes in medicine. 

Current interest in Cu complexes is stemming from their potential use as antimicrobial, antiviral, anti-inflammatory, antitumor agents, enzyme inhibitors, or chemical nucleases. Markedly, the biochemical action of Cu complexes with non-steroidal anti-inflammatory drugs (NSAIDs) has been studied [8]. Numerous Cu(II) complexes of NSAIDs showing enhanced anti-inflammatory and antiulcerogenic activity, as well as reduced gastrointestinal toxicity compared to the uncomplexed drug, have been prepared and structurally characterized [8]. They comprise a class of potential anti-inflammatory drugs with reduced side effects, and their mode of action is attributed to their marked superoxide- dismutase- (SOD-)mimetic activity. Other studies have concentrated on the potential chemotherapeutic properties of copper-based compounds [9, 10]. Moreover, several authors have brought to attention the antiviral and antibacterial activity of Cu(II) complexes. For instance, it was shown that the infectivity of influenza A virus is reduced after exposure on copper surfaces [11]. The mechanism of this process is only partly understood, but it has been speculated the degradation of the viral nucleic acid takes place after the intervention of copper ions. In addition, the study and development of Cu complexes could be helpful in the design and production of antiviral and antibacterial materials, able to deactivate HIV or H1N1 viruses [12] and antibiotic-resistant bacteria, respectively. Towards this direction, a method of producing copper-impregnated materials that possess broad-spectrum antimicrobial properties has been reported [13].

Despite the fact that the action of copper in humans has been intensively studied, the clinical picture of copper status is not always so straightforward, and less is known about the role of copper complexes in medicine. Yet it is evident that such compounds could be very important in medicinal procedures, and their role has probably been underestimated. The purpose of the present work is to review the published data in reference to the multiple physiological activities of copper, with particular focusing on the biochemical action of copper complexes and their applications in medicine. Moreover, the importance of the existing biomarkers of copper status will be evaluated.

## 2. Copper in Human Health and Disease

### 2.1. Copper Homeostasis

Copper in food (organic copper) is processed by the liver and is transported and sequestered in a safe manner. Inorganic copper, such as that in drinking water and copper supplements, largely bypasses the liver and enters the free copper pool of the blood directly. This copper is potentially toxic because it may penetrate the blood/brain barrier [1]. 

About 50% of the average daily dietary copper of around 25 *μ*mol (1.5 mg) is absorbed from the stomach and the small intestine. Absorbed copper is transported to the liver in portal blood bound to albumin and is transmitted to peripheral tissues mainly bound to ceruloplasmin and, to a lesser extent, albumin. The liver contains 10% of the total body content of 1200 *μ*mol (80 mg). Excess copper is excreted in bile into the gut, and the faecal copper output (12.5 *μ*mol/24 h) is the sum of unabsorbed dietary copper and that reexcreted into the gut [14]. Cu homeostasis is regulated by alterations in both the absorptive efficiency and biliary excretion in the gut. At low and high intakes, the efficiency of absorption is regulated up and down, respectively, but is predominantly controlled via endogenous excretion [15].

Copper is incorporated into a number of metalloenzymes involved in hemoglobin formation, drug/xenobiotic metabolism, carbohydrate metabolism, catecholamine biosynthesis and the cross-linking of collagen, elastin, and hair keratin as well as in the antioxidant defense mechanism [1]. Moreover, copper-dependent enzymes, such as cytochrome c oxidase, superoxide dismutase, ferroxidases, monoamine oxidase, and dopamine *β*-monooxygenase, function to reduce reactive oxygen species (ROS) or molecular oxygen [1]. Symptoms associated with copper deficiency in humans include normocytic, hypochromic anemia, leukopenia, and osteoporosis. Copper deficiency is rarely observed in the general population [16].

## 3. Copper and Human Health Disorders

### 3.1. Oxidative-Stress-Related Disorders

Although copper homeostatic mechanisms play an important role in the prevention of copper toxicity, exposure to excessive levels of copper can result in a number of adverse health effects including liver and kidney damage, anemia, immunotoxicity, and developmental toxicity [17]. Many of these effects are consistent with oxidative damage to membranes or macromolecules. Given the capacity of copper to produce large amounts of reactive oxygen species (ROS), an excess of Cu could result in oxidative-stress-related health disorders, many of which can be linked partially to its redox reactivity. Copper has been suggested to facilitate oxidative tissue injury through a free-radical-mediated pathway analogous to the Fenton reaction [18]. By applying the electron spin resonance (ESR) spin-trapping technique, evidence for copper-mediated hydroxyl radical formation *in vivo* has been obtained [19, 20]. ROS are produced through a Fenton-type reaction as follows:

(1) $$\begin{array}{c} \text{LCu}\left(\text{II}\right)+{\text{H}}_{2}{\text{O}}_{2}\to \text{LCu}\left(\text{I}\right)+•\text{OOH}+{\text{H}}^{+}\\ \text{LCu}\left(\text{I}\right)+{\text{H}}_{2}{\text{O}}_{2}\to \text{LCu}\left(\text{II}\right)+•\text{OH}+{\text{OH}}^{-} \end{array}$$

where L = organic ligand.

Similar to Cu toxicity, Cu deficiency also affects, directly or indirectly, the components of the oxidant defense system and as a result increased ROS and oxidative damage to lipid, DNA, and proteins have been observed in human cell culture models or clinical syndromes of severe copper deficiency [6, 21]. Copper could act as a “double-edged sword” by inducing DNA damage and also by inhibiting their repair [22]. Additionally, copper can bind directly to free thiols of cysteines resulting in oxidation and subsequent crosslinks between proteins leading to impaired activity [23].

### 3.2. Aceruloplasminemia

Ceruloplasmin is a copper-containing glycoprotein produced in the liver that binds about 95% of the copper in serum. This glycoprotein presents ferroxidase activity and catalyzes the conversion of ferrous to ferric iron which is then transferred to transferrin. A total absence of circulating serum ceruloplasmin (aceruloplasminemia) could lead to ferrous iron abundance within both the reticuloendothelial system and parenchymal cells [24]. It is noteworthy that hereditary ceruloplasmin deficiency (or aceruloplasminemia) is an autosomal recessive disorder altering iron metabolism. It is accompanied by mutations of the ceruloplasmin (*Cp*) allele on chromosome 3q [16]. Manifestations of aceruloplasminemia at the clinical level are diabetes mellitus, retinal pigmentary degeneration, dystonia, extrapyramidal signs, cerebellar ataxia, and dementia. Histopathologic studies have presented significant agglomeration of iron in the liver, pancreas, retina, and central nervous system. Although the pathogenesis of brain damage in aceruloplasminemia is currently not fully understood, it is well recognized that iron-mediated oxidative stress could be implicated in neuronal cell death [25].

### 3.3. Wilson's Disease (WD)

Wilson's disease is an autosomal recessive disease of copper metabolism of which the primary genetic defect is in ATP7B gene [26]. The biological role of ATP7B gene is to encode a copper-transport protein located at the trans-Golgi network and to transfer Cu into the secretory pathway for both annexation into ceruloplasmin and excretion into the bile [27]. A major contribution to pathophysiology of Wilson's disease is Cu-mediated oxidative damage, activation of cell death pathways, and eventual leakage of copper into the plasma pool, which ultimately results in the accumulation of excess copper in extrahepatic tissues. Notably, the hepatic Cu overload associated with WD is histopathologically characterized by bulgy hepatocytes, inflammation, and cytoskeletal alterations and finally leads to cirrhosis [28]. WD presents severe neurological symptoms, but when it is diagnosed in time, it can be treated with several ways including the use of chelating agents, low-Cu diets, and high levels of Zn supplements [29].

### 3.4. The  Menkes Disease (MD)

The Menkes disease is an X-linked recessive disorder caused by defects in a gene that encodes a copper-transporting ATPase (ATP7A) [30]. In humans, the ATP7A gene product functions as an intracellular pump to transport copper into the trans-Golgi network for incorporation into copper-requiring enzymes including dopamine-*β*-hydroxylase (DBH) and also mediates copper exodus from cells. Copper uptake and excretion by the liver are normal in MD as well as copper enzyme levels, but the absorption of copper in the gastrointestinal tract is severely impaired. The significantly decreased intestinal absorption of copper results in a shortage of exchangeable copper followed by a deficiency of cuproenzymes with important role in the developmental level [31]. It should be emphasized that the uptake by peripheral tissues is normal; however, excretion and intracellular copper trafficking are disrupted by mutations in the ATP7A gene. As a result of impaired copper efflux, peripheral tissues in MD patients tend to accumulate copper in the form of copper metallothionein. At the clinical level, MD is characterized by progressive neurological impairment and death in infancy. Because of the block in intestinal absorption of copper, the major clinical impact is from copper deficiency in the brain of the developing fetus, leading to severe brain damage [16].

### 3.5. Alzheimer's Disease (AD)

Alzheimer**'**s disease is the most common form of dementia with progressive patterns of cognitive and functional impairments. Increased levels of copper in cerebrospinal fluid accompanied by normal plasma copper concentrations in patients with AD have been found [32], while other researchers have reported elevated free copper plasma levels in AD [1]. In a rabbit model of AD, addition of trace amounts of copper (0.12 ppm) to the drinking water greatly exacerbated the brain AD pathology and loss of cognition. [33]. Moreover, a community-based prospective study suggested that high dietary intake of copper in conjunction with a diet high in saturated and trans fats may be associated with accelerated cognitive decline [34]. 

 A prominent possible involvement of copper in AD could be via its interaction with amyloid precursor protein and *β*-amyloid peptide in the self-aggregating plaques and neurofibrillary tangles, characteristic of AD, which may contribute to the pathogenesis of this disorder via cellular oxidative stress [35]. Copper ions could induce the aggregation of amyloidogenic peptide and the production of ROS which oxidize the *β*-amyloid peptide [1, 36]. Additionally, current data reveal that there is a relationship between copper metabolism in Alzheimer**'**s and prion diseases, but the precise molecular mechanism is presently unknown [37]. Nevertheless, recent investigations revealed that copper prevents both the formation and the accumulation of *β*-amyloid plaques *in vitro* [38]. In addition, it has been demonstrated that excess copper inhibits *β*-amyloid peptide production, while copper deficiency can elevate *β*-amyloid peptide secretion by either influencing amyloid precursor protein cleavage or by inhibiting its degradation [39]. It should be emphasized that Cu deficiency, similar to Cu toxicity, also results in higher ROS production and oxidative damage to proteins [6]. Although many questions remain unanswered, it is widely accepted that in AD there is an abnormal brain copper distribution with accumulation of copper in amyloid plaques and a deficiency of copper in neighbouring cells [39]. However, further investigations are needed to fully understand the role of Cu ions in AD.

### 3.6. Inflammation

Ceruloplasmin acts as an acute-phase reactive protein to stress and trauma conditions. As a consequence, elevated copper concentrations have been found in response to inflammation, infection, and various chronic diseases, such as arthritis. Serum copper levels are higher than normal in varied inflammatory diseases in humans [40]. The higher levels of ceruloplasmin are accountable for the increased serum copper in the preceding conditions. Moreover, the anti-inflammatory results of copper have been shown in humans [40]. On the other hand, the acute or chronic inflammation actuates changes on the metabolism of copper, which contribute to altered serum and tissue levels [41]. The increase of serum copper in inflammation could be due to the increase of ceruloplasmin, which is an acute-phase protein. It is well recognized that the role of ceruloplasmin in arthritis is to neutralize free oxygen radicals, mainly anion superoxide, in an attempt to stop the process of turning chronic [42, 43]. 

### 3.7. Cancer

Increased ceruloplasmin and copper levels in various tissues have been linked to cancer progression [44]. Ceruloplasmin contributes about 90% of serum copper, which is then elevated secondarily. Moreover, copper deficiency has been evaluated as an anticancer strategy even though clinical studies have not been especially encouraging [45]. While the precise role of copper in cancer development is presently not known, its involvement through ROS production in oxidative stress is possible. Recently, it was shown that copper proteins are associated with metabolic changes in cancer cells [10] and most importantly play a significant role in angiogenesis by stimulating proliferation and migration of human endothelial cells [10].

## 4. Biomarkers for Copper Status in Clinical Practice

Although several molecules-indicators have been proposed and used so far, a sensitive and specific Cu status biomarker has yet to be identified [46, 47]. To be considered as a functional index of Cu status, the chosen marker must (a) respond sensitively, specifically, and predictably to changes in the concentration and/or supply of readily available (and potentially toxic) copper, (b) be accessible for measurement and measurable, and (c) impact directly on health [17]. It is estimated that less than 5% of the total copper concentration circulates independently of binding proteins such as ceruloplasmin. The concentration of protein-free copper is very low relative to the total serum copper concentration, and therefore a clinically significant change in the free copper concentration may not be detected through measuring total copper concentrations in serum alone. Because copper is most toxic in the unbound form, measurement of unbound copper in circulation would theoretically be the most direct laboratory test for detecting potentially toxic copper overload [48]. For these reasons, a single index, such as total copper concentration in serum (determined by several standardized methods, including atomic absorption spectrometry (AAS), inductively coupled plasma emission spectroscopy (ICP), and colorimetric methods applied to autoanalyzers), is inadequate for assessing the total body copper nutriture of an individual and must be supported by collaborating evidence [49]. Usually, immunoreactive or enzymatically measured ceruloplasmin (Cp) levels are used to evaluate the body storage of copper. Cp levels are a good reflector (or biomarker) to keep copper status in the right therapeutic window, avoiding clinical copper deficiency. The enzymatic Cp/immunoturbidimetric Cp is a very useful ratio as index of copper status rather than Cp enzymatic activity or Cp concentration alone [50]. Additionally, interleukin-2 secretion from lymphocytes, neutrophil function, phenotypic profiles of lymphocyte subsets, and response of lymphocytes to T cell mitogens have been proposed [17, 51] as immunological biomarkers of marginal copper status.

The specific activity of copper-containing enzymes in blood cells, such as erythrocyte superoxide dismutase and platelet or leukocyte cytochrome c oxidase, may be a better indicator of metabolically active copper stores than the serum concentration of copper or ceruloplasmin, since the enzyme activities are sensitive to changes in copper stores but are not sensitive to factors not related to copper nutriture [49]. 

Despite the fact that the “ideal” index of copper status in adult humans has not been established yet, certain novel potential biomarkers that could be analyzed to screen for copper deficiency are currently under evaluation. Dopamine-*β*-hydroxylase (DBH), a cuproenzyme, plays a role in the production of noradrenaline. In copper deficiency, DBH activity may be lower, inducing higher ratios of dopamine to norepinephrine [52]. Peptidylglycine *α*-amidating monooxygenase and lysyl oxidase are both cuproenzymes whose activity or gene expression may be influenced by copper deficiency [46]. Moreover, there are also a number of potential biomarkers of copper overload. High levels of copper are accumulated in the liver where the excess of copper can lead to damage. Through nonspecific, the hepatic aminotransferase enzymes are biomarkers for liver abnormality due to copper overload. Urinary *β*2-microglobulin could additionally be an assisting marker of copper excess [53]. Copper chaperon for superoxide dismutase (CCS) has been proposed [46] as a sensitive and accurate biomarker since it reflects both deficiency and excess states of Cu. CCS levels in blood erythrocytes and white cells are determined with specific antibodies and have been shown to vary inversely proportional to Cu status [54]. In a recently published study, a volumetric analytical method has been proposed to quantify copper complexes [55].

## 5. Copper Complexes as Potential Therapeutic Agents

### 5.1. Binary Cu(II) Complexes

A number of Cu(II) chelate complexes that exhibit cytotoxic activity through cell apoptosis or enzyme inhibition have been reviewed [56]. Such complexes containing bi-Schiff bases as ligands are effective in reducing tumor size, delaying of metastasis, and significantly increasing the survival of the hosts. Chelates of curcuminoids show significant reduction of solid tumor volume in mice (*P* < .001), while complexes of pyridine-2-carbohidrazide derivatives inhibit the expression of c-Src, a nonreceptor tyrosine kinase, which plays a significant role in growth-mediated signaling pathway, thus showing cytotoxicity against colon cancer cell lines. Similarly Cu(II) chelates of salclaldoxime and resorcylaldoxime [57] are potent antiproliferative agents, exhibiting strong cytotoxic effects comparable to that of adriamycin, by inducing cell cycle arrest and apoptosis. Their action may involve the inhibition of the enzyme topoisomerase II activity, by preventing dimer formation of the enzyme and its interaction with DNA [58]. The diverse biological activity of these complexes compared to one of the widely used platinum anticancer drugs cisplatin, indicates different mechanism(s) of action, which have not been yet resolved. It is likely that copper complexes interact with enzymes and inhibit vital cell functions, rather than interact with DNA and induce crosslinks. 

Binary Cu(II) complexes with a variety of aromatic molecules coordinated through N, S, or O donor atoms have been synthesized and tested for biological activity. Some of them are presented in Table 1. 

The complex 2,6-bis(benzimidazo-2-yl)pyridine copper(II) chloride has been shown to exhibit metalloprotease activity [59]. It binds to bovine serum albumin causing site- specific cleavage of the protein when the system is incubated in atmospheric conditions. This is believed to take place through binding and activation of molecular oxygen by the metal. Complexes of carboxamidrazones exhibit enhanced antiproliferative activity against B16F10 mouse melanoma cells [60]. It is suggested that the combination of Cu(II) with carboxamidrazone ligands may facilitate intracellular transportation and block estrogen receptors. Coordination of N^6^-substituted adenines to Cu(II) also results in enhanced cytotoxic activity against various forms of human cancer [61]. In this case as well, the resulting Cu(II) complexes inhibit cyclin-dependent kinases.

Though copper is an essential cofactor for tumor angiogenesis processes, several Cu(II) binary complexes have been reported to function as proteasome inhibitors, inducing apoptosis in various types of human cancer cells. In such complexes, described as “organic copper compounds” [2], the metal is coordinated either to neutral heteroatomic molecules such as phenanthroline or to anionic organic ligands such as 8-hydroxyquinolinate, pyrrolidine dithiocarbamate, or (pyridine-2-ylmethylamino)methyl phenolate. It is noticeable that the free ligands themselves are not efficient inhibitors, and complex formation is necessary for the transportation of copper ions through the cell membrane, in order to achieve proteasome inhibition [64]. This seems to be the result of the increasing lipophilicity of the metal upon ligand coordination. Similar zinc complexes have been found to be less-efficient proteasome inhibitors [65].

Copper deficiency can inhibit angiogenesis, thus preventing the growth of tumor cells or an inflammation to spread. In this regard the control of copper levels can be potentially used as a strategy in the therapy of cancer or against neurodegenerative diseases [68]. The role of copper in angiogenesis processes is not yet understood, and further research is needed for this purpose. In order to enhance biological activity, it has been a common practice to synthesize Cu(II) complexes of biologically active ligands. Thus complexes of thiosemicarbazones have been extensively studied in efforts to synthesize efficient anticancer drugs [9]. Such complexes were also found to inhibit enzymatic activity and induce cell apoptosis [10] such activity is usually correlated with anticancer drug validity. Similarly Cu(II) complexes of nitrophenone thiosemicarbazones have been found to exhibit significant antitrypanosomal activity *in vitro* [63]. A number of binary Cu(II) complexes of 2-carboxaldehyde thiosemicarbazone derivatives [62], quinolone derivatives [66], and benzenesulfonamide derivatives [67] that have been synthesized, structurally characterized, and tested for biological activity are cited in Table 1.

In contrast to the large number of Cu(II) complexes that have been synthesized as potential drugs, Cu(I) ones are sparse due to the lower stability of the +1 redox state and their propensity towards oxidation to Cu(II) compounds. Binary Cu(I) complexes of the formulae CuLCl, with L = N,N′-disubstituted thioureas and [CuL_4_]^+^ with L = 1,3,5-triaza-7-phosphaadamantane exhibit moderate cytotoxicity against various human cell lines [69].

### 5.2. Ternary Cu(II) Complexes

Numerous mixed ligand complexes that combine one or two bidentate N,N- and O,O-coordinated ligands have been synthesized and tested for biological activity. Chemical formula of representative Cu(II) ternary complexes together with the coordination mode of various ligands are cited in Table 2. Complexes of the type [CuLL′]^+^, where L = N,N-chelate such as phenanthroline or 2,2′-bipyridine and L′ = N,N- or N,O-chelate such as acetylacetonate or glycinate (see Table 2), are known as casiopeinas [70]. They exhibit significant antineoplastic activity *in vitro* and *in vivo*, against a variety of tumor cell lines. 

They interact with mitochondria of both healthy and tumor cells, inhibiting oxidative phosphorylation and respiration [70]. In addition they exhibit high affinity towards DNA binding as well as nuclease activity towards plasmid, genomic, and internucleosomal DNA. Degradation is achieved through the production of reactive oxygen species (hydroxyl radicals) [78]. The complex of o-iodohippuric acid (see Table 2) exhibits both antitumor and nuclease activity. Inhibition (up to 35%) of lung adenocarcinoma (A549) cell growth takes place within 24 hours when the complex was administered in a concentration of 75 nM. Healthy cells were not affected under the same conditions [71].

A number of mixed ligand complexes with Schiff bases and 2-amino-2-thiazoline, included in Table 2, have been reported to show significant anti-inflammatory, antibacterial, and anticancer activity against various cell lines [72]. The biological activity of these complexes does not correlate with their lipophilicity. Several quinolone mixed ligand complexes that have been synthesized and structurally characterized [73–76] are also summarized in Table 2. They have been found [55] to exhibit *in vitro* antimicrobial activity as well as concentration-dependent cytotoxicity against human leukemia cells, with the sparfloxacin complex being the most potent. These complexes were also found to bind in calf-thymus DNA in an intercalate mode.

A few Cu(I) complexes have been synthesized and tested *in vitro* as potential anticancer drugs. Mixed ligand Cu(I) complexes of triazolylborate and alkyl- or aryl-phosphines have been found to be effective against A549 adenocarcinoma cells that are resistant to the widely used anticancer drug, cisplatin [79].

## 6. Biological Activity of Copper Complexes

Biological screening results of representative binary and ternary copper complexes are shown in Table 3.

Metalloprotease activity of the complex 2,6-bis(benzimidazo-2-yl)pyridine copper (II) chloride was found with sodium dodecyl sulfate polyacrylamide gel electrophoresis (SDS-PAGE) with bovine serum albumin in the presence of oxygen. Albumin undergoes site-specific cleavage with the resultant formation of four fragments of molecular weight 49, 45, 22 and 17 kDa [59]. Bridged Cu(II) complexes of 6-(methoxybenzylamino)purines show antioxidant activity both *in vitro* and *in vivo. *Values of IC_50 _ = 0.253 up to 1.250 *μ*M were reported for the superoxide-dismutase- (SOD-)mimic activity for the Cu complex *in vitro* compared to IC_50_ = 0.480 *μ*M of the native bovine Cu,Zn-SOD enzyme, used as a standard. The pretreatment of mice with Cu complexes *in vivo* led to the complete elimination of cytotoxic attack of alloxan-induced *diabetes* and its free radical metabolites (cytoprotective effect) [80]. Copper complexes that exhibit high SOD-like activity are potent drugs for prion diseases since such activity is correlated with antisprion activity. It is known that prion proteins can bind Cu(II) ions with high specificity as they possess a number of copper sites. Moreover, in the development of prion disease, copper may modulate the rate of protein misfolding [81].

Bis(5-nitrofuran-2-carboxaldehyde thiosemicarbazone) copper (II) chloride has been reported [62] to exhibit *in vitro *antiamoebic activity against *HK-9* strain of *Entamoeba histolytica* with IC_50_ value lower than the corresponding metronidazole, the drug of choice for amoebiasis (IC_50_ = 0.38 *μ*M and IC_50_ = 0.34 *μ*M versus IC_50_ = 1.81 *μ*M of metronidazole). These results indicate that the metallated thiosemicarbazone may be a lead molecule in inhibiting growth of *E. histolytica*.

Bis(4-nitroacetophenone thiosemicarbazone) copper (II) complexes exhibit antitrypanosomal activity *in vitro* against the epimastigote form of *Trypanosoma cruzi.* They presented less ID_50_ value (the dose that inhibits 50% of *T.cruzi *growth) than their corresponding thiosemicarbazones (ID_50_ = 0.056 *μ*M for the best anti-*T.cruzi* copper complex versus ID_50_ = 0.28 *μ*M) [63].

Pyrrolidine dithiocarbamate complexes of Cu(II) were reported to cause the inhibition of the proteasome (cancer cells are more sensitive to proteasome inhibition than normal cells) *in vitro* against LNCaP prostate cancer cells using two assays as indicators of proteasome inhibition: (a) the chymotrypsin-like activity assay and (b) a Western blotting analysis performed by antiubiquitin antibody for the accumulation of ubiquitinated proteins [82]. The copper complexes resulted in low levels of the proteasomal chymotrypsin-like activity and the accumulation of ubiquitinated proteins. In contrast, copper or the free ligand alone, was incapable of inhibiting the proteasome. In addition pyrrolidine dithiocarbamate-copper complexes were found [82] to suppress the proliferation of BE(2)C cells, a human neuroblastoma cell line, with an IC_50 _  = 8.0 *μ*M, which is more potent than cisplatin (IC_50 _ = 80 *μ*M). A chelate Cu(II) complex of N-pyridinobenzamide-2-carboxylic acid (PBCA) has been tested *in vivo* for anti-inflammatory activity [83]. The complex shows a greater decrease (26.5%) in inflammation compared with free PBCA (20.2%) against the cotton pellet granuloma pouch test in rats (the subcutaneous implantation of a cotton pellet into a rat results in the formation of a granuloma at the site of the implant).

The neutral bimetallic complex [Cu_2_(ibuprofen)_4_] with the bridged ligand 2-(4-isobutylphenyl)propionate (ibuprofen) exhibits antiulcerogenic *in vivo *activity as estimated for gastric irritation in rats [84]. Rats treated with ibuprofen exhibited a lesion index of 597 ± 43, while markedly lower lesion indices 290 ± 31 were observed for rats treated with the copper-ibuprofenato complex. The copper complex was more effective in the protection of severe intensity ulceration than the oral administration of the parent drug (free ibuprofen). The Cu (II) chelates with quinolone derivatives cited in Tables 1 and 2 exhibit antibacterial activity *in vitro* against *Staphylococcus aureus* ATCC25923 and *Staphylococcus aureus* ATC57. The best antibacterial copper complex [66] shows a minimum inhibitory concentration (Andrews 2001) MIC = 0.5 *μ*g/mL versus free ciprofloxacin hydrochloride with MIC = 1 *μ*g/mL. 

Aiming at the synthesis of potent anticancer drugs, binuclear copper(II) complexes of pyridyl-diamines, as well as mixed-ligand acetylacetone/quinoxaline complexes exhibiting nuclease and apoptosis-inducing activity, have been reported recently [77, 85]. In addition the antitumor activity of Schiff-base copper(II) complexes has been investigated [86, 87]. The evaluation of a new thiosemicarbazone Cu(II) chelate was reported to induce tumor growth inhibition both *in vitro* and *in vivo*, through oxidative/endoplasmic reticulum stress [88]. 

## 7. Conclusions

Developing an integrated picture for the role of copper and its complexes in medicine is a challenging task that awaits further exploration. Copper ions are considered as multifunctional participating in a broad spectrum of intracellular processes under normal and pathologic conditions. However, many questions remain unanswered. Further experimental and clinical studies would aid at unraveling their prominent activities, thus discovering effective Cu biomarkers and generating new options for early intervention in copper-related health disorders. Copper complexes described in the present work show a diverse *in vitro* biological activity, ranging from antibacterial and anti-inflammatory to cytostatic and enzyme inhibitory. At molecular level such complexes interact directly with proteins and DNA, leading to dysfunction and cleavage of the macromolecular structure, or indirectly producing ROS that attack and degrade biomolecules. 

Since DNA is a potent target of cytostatic drugs, the effect of copper compounds on DNA functionality is very important. The ability of Cu(II) complexes to bind to DNA and exhibit nuclease activity in the presence of reducing agents is well established [89]. DNA degradation is believed to take place through a Fenton-type reaction in which ROS are produced. The type of organic ligands in such copper complexes seems to affect and regulate their activity. They (a) neutralize the electric charge of the copper ion, (b) increase the lipophilicity of the complex facilitating transport through cell membrane, and (c) intercalate to DNA or interact noncovalently with proteins. The effect of ligand chelation may also be of importance for the biological activity of the complexes, whose exact role has not been elucidated yet. Spatial geometry of the complex and the structure of the ligand influence to a lesser extent the activity of the complex. The biological activity of the ligand is usually increased upon complex formation, but evidence of a synergic effect between the metal and the organic ligand is still lacking.

In conclusion, novel treatment options that interfere with copper complexes have been proposed in experimental systems, albeit their effectiveness in clinical practice remains to be further investigated. The great pressure for producing new effective treatment options in medicine should not surpass the necessity for careful, rationally designed randomized studies evaluating the most promising copper complexes as therapeutic pharmaceuticals.

## Figures and Tables

**Table 1:** Binary Cu(II) Complexes that exhibit biological activity.

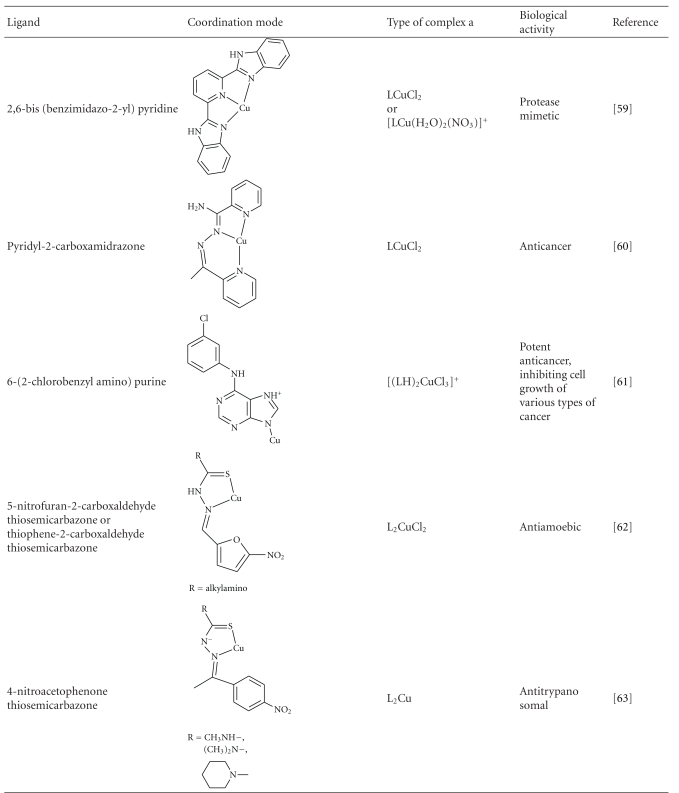 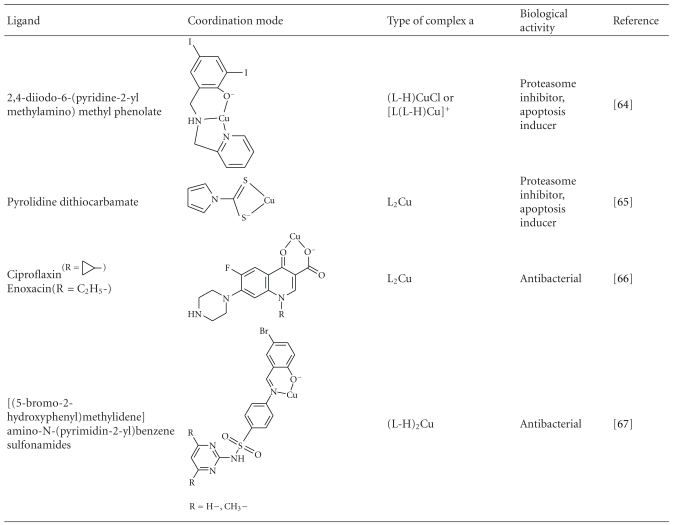

^a^(LH) denotes protonated ligand; (L-H) denotes deprotonated ligand.

**Table 2:** Coordination modes of organic ligands in ternary Cu(II) complexes.

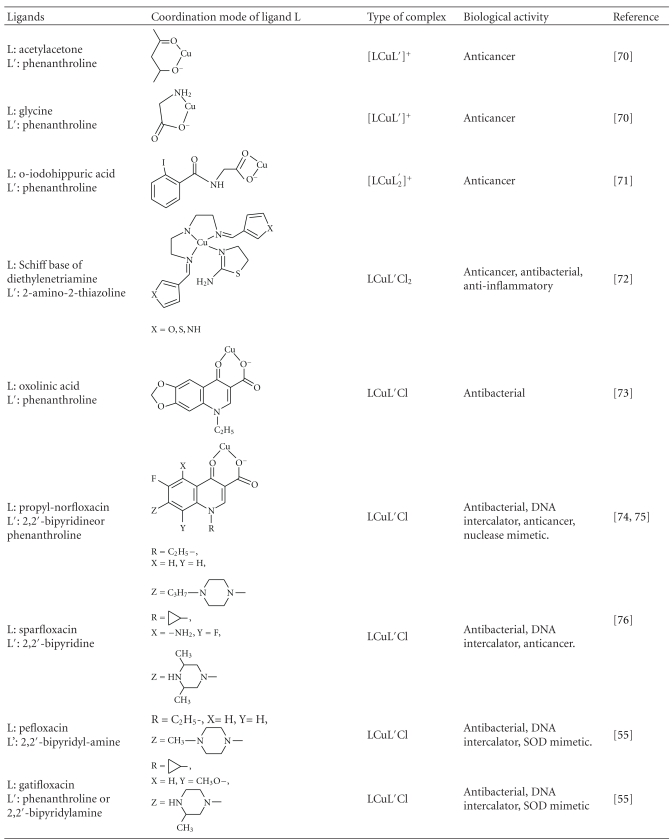 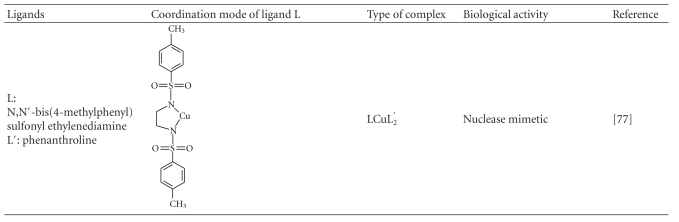

**Table 3:** Inhibitory effects of selected copper complexes.

Complex	Biological *in vitro* activity		IC_50 _^ a,b^ (*μΜ*)	Reference
Bis(4-nitroacetophenone thiosemicarbazonat) copper(II)	Anti-trypanosomal	*Trypanosoma cruzi*	0.056 ^c^ (0.28)^c^	[63]
Bis(5-nitrofuran-2-carboxaldehyde-N_4_-ethylpiperidine thiosemicarbazone) copper(II) chloride	Antiamoebic	HK-9 strain of *Entamoeba histolytica *	0.34(2.68)	[62]
Bis(thiophene-2-carboxaldehyde-N^4^-methyl benzyl thiosemicarbazone) copper(II) chloride	Antiamoebic	HK-9 strain of *Entamoeba histolytica *	0.21(1.21)	[62]
(2-acetylpyridine)pyridine-2-carboxamidrazone copper(II) chloride	Antiproliferative	Mouse melanoma cell line B16F10.^ d^	6.8	[60]
Cu_2_(*μ*-L)_2_(*μ*-Cl)_2_L_2_Cl_2_L= 6-(2-chlorobenzylamino) purine	Antitumor	Mouse melanoma B16FO	8.2(85)	[61]
		Human malignant melanoma G361	20.0	[61]
			(95)	
		Human osteogenic sarcoma HOS	23 (>100)	[61]
		Human breast adenocarcinoma MCF7	24(>100)	[61]
Tetra(N-methyl-1,3,5-triaza-7-phosphaadamantane copper(I) tetrafluoro borate	Antitumor	Cervix tumor (HeLa)	7.6	[69]
		Colon tumor (HCT-15)	19.2	[69]
		Lung tumor (A549)	8.5	[69]
		Melanoma (A375)	16.4	[69]
Bis(triphenylphosphine), dihydridobis(3-nitro-1,2,4-triazolyl)borate copper(I)	Antitumor	Promyelocytic leukemia (HL60)	4.8	[79]
		Cervix carcinoma (A431)	6.7	[69]
		Lung tumor (A549)	1.5	[69]
		Melanoma (A375)	16.4	[69]

^a^Values refer to the most active copper (II) complex tested. Half- maximal inhibitory concentration (IC_50_) is a quantitative measure of the effectiveness of a compound in inhibiting a biological function as it indicates how much of a particular substance is needed to inhibit a given biological process.

^b^Values in parenthesis refer to the free ligand.

^c^These values refer to inhibitory dose (ID_50_): dose that inhibits 50% of *Trypanosoma cruzi* growth.

^d^The exponentially growing cells were counted by haemocytometer using the Trypan blue exclusion method to quantify cell viability.
